# The Contribution of the Cerebellum to Cognition in Spinocerebellar Ataxia Type 6

**DOI:** 10.3233/BEN-2010-0265

**Published:** 2010-08-16

**Authors:** Freya E. Cooper, Manon Grube, Kelly J. Elsegood, John L. Welch, Thomas P. Kelly, Patrick F. Chinnery, Timothy D. Griffiths

**Affiliations:** ^1^Institute of NeuroscienceNewcastle UniversityNewcastleUK; ^2^Trafford Child & Adolescent Mental Health ServiceManchesterUK; ^3^Department of NeuropsychologyNewcastle UniversityNewcastleUK; ^4^Department of NeurologyNewcastle UniversityNewcastleUK

**Keywords:** SCA-6, cerebellum, cognition, neurodegeneration, executive functions

## Abstract

This study sought evidence for a specific cerebellar contribution to cognition by characterising the cognitive phenotype of Spinocerebellar Ataxia Type 6 (SCA-6); an autosomal dominant genetic disease which causes a highly specific late-onset cerebellar degeneration. A comprehensive neuropsychological assessment was administered to 27 patients with genetically confirmed SCA-6. General intellectual ability, memory and executive function were examined using internationally standardised tests (Wechsler Adult Intelligence Scale-III, Wechsler Memory Scale-III, Delis and Kaplan Executive Function System, Brixton Spatial Anticipation test). The patient group showed no evidence of intellectual or memory decline. However, tests of executive function involving skills of cognitive flexibility, inhibition of response and verbal reasoning and abstraction demonstrated significant impairment at the group level with large effect sizes. The results demonstrate an executive deficit due to SCA-6 that can be conceptualised as parallel to the motor difficulties suffered by these patients: the data support a role for the cerebellum in the regulation and coordination of cognitive, as well as motor processes that is relevant to individual performance.

## 1. Introduction

The cerebellum is traditionally viewed as a servomechanism for motor control that assists in the event of departure from a planned course. Recent evidence suggests an additional role in higher-order cognitive processes.

Functional imaging studies of normal subjects have documented the activation of the cerebellum as part of neural networks responsible for motor planning, working memory, executive and spatial functions, language and emotional processes e.g. [5,9,10,12,19,38]. Based on a meta-analysis of such imaging studies, Stoodley and Schmahmann [59] argue for a functional dissociation between the anterior and posterior cerebellum in sensorimotor vs. cognitive processes. The aforementioned studies argue for a role for the cerebellum in non-motor cognitive tasks as part of a wider network of areas including the frontal and parietal cortices. Anatomical studies document the connections between the cerebellum and the prefrontal and parietal areas via the ventral dentate nucleus [15,37,43], that are separate from the projections to primary motor and pre-motor areas via the dorsal dentate nucleus. These projections suggest distinct anatomical bases for the contribution of the cerebellum to cognitive and motor processes. The underlying computational process within the cerebellum might be similar in both cases: a parallel can be drawn between motor correction and smoothing by the cerebellum to produce a continuous and coordinated movement, and the effect of the cerebellum on cognition [43]. This parallel is most compelling in the case of executive function, where the central executive can be

ISSN 0953-4180/10/$27.50 © 2010 – IOS Press and the authors. All rights reserved compared to a servomechanism for motor control that acts as a corrective mechanism in the event of departure from a planned outcome.

Studies of patients with cerebellar damage allow a test of the cognitive functions for which the cerebellum might be an obligatory substrate. Performance in working memory [13,23,34,35,42,48,57], visuospatial memory [16,22,23,49,62] and general memory including both immediate and delayed memory [23,39, 42] has been found to be significantly poorer in patients with cerebellar damage than in controls. Subtle deficits in language, separate from dysarthria, have been noted in patients, such as poor grammatical judgement in speech [29], word finding difficulties [1], problems with reading (decoding syllable-by-syllable) and agrammatical written output [30]. In children with posterior fossa syndrome, specific damage to the vermis can lead to post operative mutism and poor language comprehension [47,49,56]. Visuospatial and constructional difficulties are also noted in these patient groups [16,41,62]. Executive functions are consistently reported to be affected in cerebellar patients, with poorer performance in tests of planning, cognitive inhibition and flexibility [22,23,39,42], sequencing [36], attention [23,39,55,62], and in tests of both verbal [22, 23,39,42] and non-verbal fluency [23]. These findings are generally reported in the context of otherwise intact intellectual abilities and cannot be explained by a generalised decline in intelligence.

Schmahmann and Sherman [52] have argued for a cerebellar cognitive affective syndrome (CCAS) in patients with cerebellar damage that is unrelated to the extent of dysarthria or motor problems suffered by patients. They argue that damage to this area leads to a characteristic set of deficits consisting of: i) widespread executive dysfunction including deficits in planning, set-shifting, abstract reasoning and verbal fluency; ii) impaired spatial cognition, also involving visuospatial memory; iii) personality and affective change characterised by inappropriate and childlike behaviour and flattened affect; and finally iv) linguistic difficulties including deficits in naming, grammar and prosody.

Previous clinical studies have examined heterogeneous groups of patients with congenital and acquired lesions [13,16,17,22,23,30,35,36,49,56,62], that did not affect the cerebellum in an identical way across the group, and which also involved extracerebellar structures. Such studies do not allow clear inference about the obligatory role of the cerebellum in cognition. In this study we assess the cognitive deficits due to a focal cerebellar degeneration that has a stereotyped pattern of degeneration in affected individuals: Spinocerebellar Ataxia type 6 (SCA-6). SCA-6 is an autosomal dominant neurodegenerative condition caused by an extended CAG repeat mutation of the CACNA1A gene on chromosome 19p13: a locus known to code for the $\alpha$1A calcium channel subunit on the P/Q type voltage dependent calcium channels [67], which is crucial for Purkinje cell function and survival. The degeneration of the Purkinje cells in SCA-6 leads to a slowly progressing, late-onset ataxia which usually first presents after 50 years of age [53], with patients gradually losing the ability to walk about 15 years after the onset of symptoms [25]. Dysarthria, trunk and limb ataxia and occulomotor disturbances are also present in this disorder [21,25,28,31,53,54,58,61,68]. The behavioural phenotype of SCA-6 is associated with a stereotyped progression from the dorsal to the ventral parts. Atrophy is typically more marked in the vermis, but is also observed in the lateral cerebellar hemispheres [8] (See Fig. 1). This atrophy is due to Purkinje cell degeneration in the cerebellar cortex: there is usually no significant white matter loss or extra-cerebellar neuronal loss in the brainstem, cerebellar peduncles and olivary nuclei [8,21,26,40,53,54,63]. The pure cerebellar degeneration found in this disorder makes it an excellent model to investigate the possible contribution of the cerebellum to cognitive processes.

Only four studies to date have investigated cognition in SCA-6. General intellect and attention [18,20,60], verbal working memory [20,60] and visuospatial perception [18,60] are apparently intact in SCA-6 subjects. Immediate verbal memory was found to be impaired when compared to controls in one study [60], with 2 patients out of 9 being impaired in another [18]. Similarly immediate visual memory has also been found to be impaired in SCA-6 groups [18,32,60], with delayed memory in both domains preserved [18,20,32, 60]. Scores of verbal fluency were also found to be significantly lower than controls in SCA-6, and did not correlate with the degree of dysarthria suffered [32,60]. Impairments have also been found in a number tests of executive functioning: performance on tests of problem solving behaviour, conceptual reasoning and cognitive flexibility (card sorting tests) has been found to be significantly affected compared to controls [60], with other studies finding non-significant trends of consistently worse performance by patients [20,32]. In a more demanding test of cognitive inhibition (the Hayling Sentence Completion Task [7]) a deficit in performance in 4 out of 9 SCA-6 patients was found [18]. This suggests a profile of intact general intellectual abilities includ-

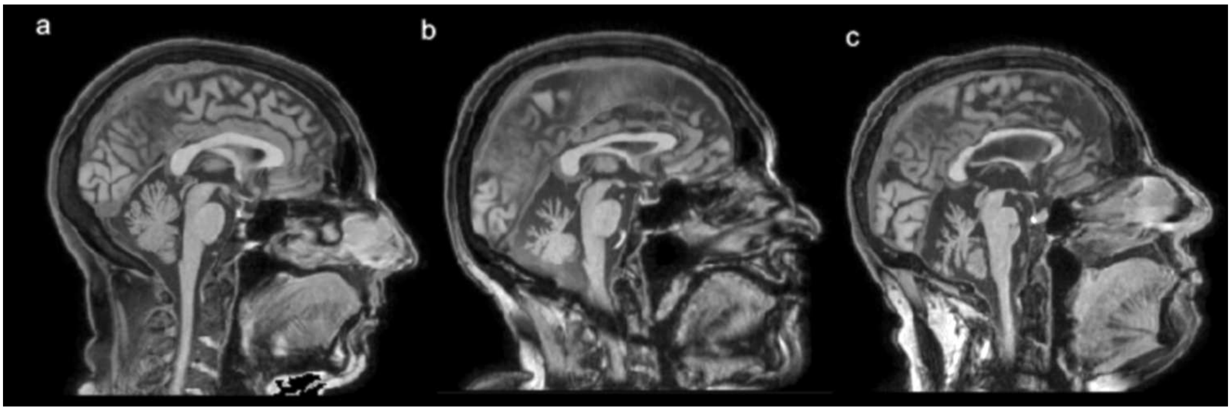

Fig. 1. MRI scans of 3 female participants with SCA6 to show progression of degeneration. a: aged 58 years, no motor symptoms at time of testing ($x = 3$); b: aged 75 years, desease duration 5 years ($x = 5$); c: aged 73 years, disease duration 8 years ($x = 4$) (MNI) coordinates.

ing attention, working memory and visuospatial perception, with mild impairment in immediate memory performance and some aspects of executive function.

The interpretation of previous studies is limited by small group sizes (9–18 patients) and absence of systematic test batteries (many used single subtests from different test packages). This makes it difficult to draw strong conclusions about the cognitive phenotype of this disease and about the contribution of the cerebellum to cognitive functions. The present study examines the largest group of SCA-6 patients to date ($n = 27$) with a systematic and comprehensive battery of current neuropsychological tests. An estimate of pre-morbid IQ was taken using the Wechsler Test of Adult Reading UK (WTAR-UK) [66]. Current intellectual abilities were tested using all subtests of the Wechsler Adult Intelligence Test-III (WAIS-III UK) which yields indices of Verbal Comprehension, Perceptual Organisation and Processing Speed as well as Verbal, Performance and Full Scale IQ scores [64]. Immediate and delayed memory were tested in both visual and verbal domains using all subtests of the Wechsler Memory Scale-III (WMS-III UK) [65]. Executive functioning was investigated using all subtests (excluding the proverb test due to unsuitability for assessing a British population) of the Delis and Kaplan Executive Function System (D-KEFS) [14] and the Brixton test of Spatial Anticipation [7].

These assessments allow a comprehensive test of the hypothesis that cerebellar dysfunction due to SCA-6 causes deficits in executive function as predicted by the CCAS [52]. We predict that the magnitude of such deficits should correlate with the severity of the motor deficit and disease progression as markers of the extent and severity of the cerebellar lesion. This is especially important in a cohort defined genetically where some subjects are unaffected or minimally affected: the critical test here is of the relationship between neuropsychological deficits and cerebellar damage rather than the presence of the abnormal gene *per se.* The tests also allowed the assessment of other deficits suggested by studies of smaller cohorts of SCA-6 patients including planning [20], set shifting [20,32,60], abstract reasoning [60], immediate memory [20,60], verbal fluency [20,32,60] and spatial cognition [18,32,60].

## 2. Methods

### *2.1. Subjects*

27 subjects with genetically diagnosed SCA-6 took part in this study: 10 male, 17 female, age range 47–79 years (mean 65 years, standard deviation 9 years). Mean age of onset of disease was 50 years (standard deviation 22 years) with mean self-reported disease duration of 9 years (standard deviation 2 years). This group had a mean of 11 years education (standard deviation 2 years). No subject reported any other neurological condition apart from their ataxia, and no subject was suffering from, or had previously suffered from, any psychiatric illness. Six subjects were taking medication to treat high blood pressure and two to treat thyroid problems. One subject was on the antidepressant Amitriptyline. Three subjects were asymptomatic at the time of testing. Subjects received travel and expenses reimbursement. This study was approved by the Newcastle and North Tyneside 2 Research Ethics Committee. All subjects gave informed consent.

### 2.2. Neuropsychological assessment

Participants were assessed using a comprehensive battery of neuropsychological tests. Most patients were assessed in their homes with the exception of four subjects who visited the laboratory. There was no discernable difference in performance between those who were tested at home and the four subjects who came to the laboratory. The assessment was carried out over 2–3 sessions each lasting less than 2 hours to limit fatigue in participants.

#### 2.2.1. Intellectual functioning

Premorbid intellectual functioning was measured using the WTAR-UK [66]. Current intellectual functioning was assessed by administering all subtests from the WAIS-III UK following the standardised administration protocol detailed in the test manual [64]. This includes measures of Verbal, Performance and Full Scale IQ, and index scales of Verbal Comprehension, Perceptual Organisation, Verbal Auditory Working Memory and Processing Speed. A difference of greater than 15 (one standard deviation) between the predicted Full Scale IQ score and current Full Scale IQ score was taken as evidence of global intellectual decline [66]. The significance attributed to such a difference is $p < 0.05$ at all ages.

#### 2.2.2. Memory

Immediate, delayed and working memory in both auditory and visual domains was investigated using all subtests of the WMS-III UK following the standardised administration protocol detailed in the test manual [65].

#### 2.2.3. Executive functioning

A number of aspects of executive functioning were examined using all subtests of the D-KEFS [14] (excluding the proverb test). This includes verbal and nonverbal tests of planning, reasoning, creativity, verbal fluency, problem solving, cognitive flexibility and inhibition. The Brixton test of Spatial Anticipation [7] was also used to assess non-verbal aspects of cognitive flexibility.

### 2.3. Motor impairment

Motor impairment was measured on a locally-developed scale of 1–10, based on four subscales measuring the severity of gait (0 = unaffected, 1 = mild, 2 = use of stick, 3 = use of stroller, 4 = use of wheelchair), limb ataxia (0 = unaffected, 1 = mild, 2 = severe), dysarthria (0 = unaffected, 1 = mild, 2 = moderate, 3 = severe) and nystagmus (0 = not present, 1 = present). The total score from these four subscales gave the measure of motor impairment used in the correlation analysis.

### 2.4. Data analysis

All neuropsychological tests used have internationally age-standardised published norms, with the single exception of the Brixton test of Spatial Anticipation. For that test, normal age-grouped data collected by Bielak and colleagues [3] were first used to convert individual SCA-6 scores to age dependent z scores. This was done using the formula:

$$\text{z} = (\text{E SCA-6} - \text{Econtrol})/\text{SDcontrol}$$

Where ESCA-6 is the individual SCA-6 error score, $\text{E},^{-}{}_{\text{control}}$ is the mean of error scores for the appropriate age-matched controls for that individual and SD-controls is the standard deviation of the age-grouped controls. For ease of comparison with the other measures, z scores were then converted to standard scores with a mean of 100 and standard deviation of 15.

Distribution of data samples was tested by the Lilliefors-corrected Kolmogorov-Smirnov test for normal distribution. Population inference was made based on one-tailed one-sample t-tests applied to normally distributed samples to test the hypothesis that the mean for the SCA-6 group was significantly lower than the control mean of 100 (WAIS-III, WMS-III and Brixton test of Spatial Anticipation) or 10 (D-KEFS subsets). The one-sample Wilcoxon ranked sign test (W) was applied to non-normally distributed samples using a control median of 100 (Verbal Working Memory and Processing Speed index scores) or 10 (motor speed subtest of Trail Making Test, empty dots subtest of Design Fluency Test, inhibition/switching subtest of Colour/Word Interference Test and initial abstraction score of the 20 Questions Test). Effect sizes for one-sample t-tests were calculated using Cohen's d [11], those for ranked based tests could not be calculated. Correlation analysis to investigate a relationship between severity of motor impairment (using the total motor impairment score obtained from the locally-developed scale described above), disease duration and all cognitive index scores was carried out using Spearman's Rho (one-tailed), as not all index scores were normally distributed. A significance level of $p = 0.05$ was used for all statistical analyses, without correction for multiple comparisons in the presence of a priori hypotheses about abnormalities within individual subtests.

Table 1
Pre-morbid and current intellectual functioning of SCA-6 group and comparison with control data

| IQ/Index scale | N | Predicted IQ mean (SD) | Mean (SD) | Statistical analysis (p-value) | Number impaired (%) |
|---|---|---|---|---|---|
| Verbal IQ | 27 | 102 (8.2) | 98 (10) | $t = -1.2$ (ns) | 2 (7.4%) |
| Performance IQ | 27 | 103 (7.2) | 97 (14) | $t = -1.0$ (ns) | 6 (22%) |
| Full Scale IQ | 27 | 102 (8.4) | 97 (12) | $t = -1.3$ (ns) | 6 (22%) |
| Verbal Comprehension | 27 | | 98 (12) | $t = -0.7$ (ns) | 4 (15%) |
| Perceptual Organisation | 27 | | 103 (14) | $t = 1.0$ (ns) | 1 (3.7%) |
| Verbal Working Memory | 27 | | Median = 96 | $W = 130$ (ns) | 3 (11%) |
| Processing Speed | 25 | | Median = 90 | $W = 42$ ($< 0.01$)* | 9 (36%) |

Mean WTAR predicted IQ and WAIS-III IQ and Index Scores with results from one-tailed one-sample t-test (t) (parametrically distributed data) and one-tailed one sample Wilcoxon test (W) (non-parametrically distributed data) to compare patient group performance with age-standardised control data with mean and median 100 and standard deviation 15. (ns denotes 'not significant', * denotes significance at the $p = 0.05$ level). Number and percentage of individually impaired patients also included (index score 15 points or more below the control mean).

## 3. Results

Most patients could complete all aspects of the test battery, however some could not complete subtests that involved a motor response due to severe limb ataxia: block design (2), picture arrangement (1), symbol search (2) (WAIS-III), spatial span (1) (WMS-III), trails (3), design fluency (4) and card sort (1) (D-KEFS). Two patients failed the screening tests of the colour/word interference subtest of the D-KEFS and so do not have a complete score. Two patients were uncooperative with the verbal paired associates task (WMS-III), and one with the 20 Questions task (D-KEFS). One patient did not take part in the D-KEFS battery. Where possible, pro-rating of subtests for these individual patients have been performed to obtain the relevant index score.

### *3.1. Definition of deficits using group and individual inference*

#### *3.1.1. Intellectual function*

At the group level, no significant difference from control data was found for scores of predicted Full Scale IQ. No difference was found between patient performance and standardised norms for current Verbal, Performance and Full Scale IQ scores or for the composite indices Verbal Comprehension, Perceptual Organisation and Working Memory (see Table 1). Processing Speed based on tasks requiring a timed motor output was unsurprisingly significantly slower in the SCA-6 patient group than the standardised normal data. Two patients (7.4% of group) showed a difference of $> 15$ ($-1$ standard deviation) between their predicted Full Scale IQ score and their current Full Scale IQ score, indicating a decline in intellectual function for these two subjects. This is comparable to the 5–9% of the normal population where this discrepancy between predicted and current IQ is found [66].

#### *3.1.2. Memory function*

No significant abnormality of scores at the group level was found in any index scale of memory function (Table 2). However 26% of individual patients could be classed as impaired on the Auditory Immediate Scale and 30% impaired on the Visual Immediate and Immediate Memory scales ($\geqslant 15$ points below the control mean of 100). 16% of the normal population achieve an index score of 85 or less, indicating an increase in number of individuals impaired in the SCA-6 group compared to the normal population.

#### *3.1.3. Executive function*

In tests of verbal and non-verbal fluency, non-verbal conceptual reasoning (Free Card Sort), verbal deductive reasoning (Word in Context task), and non-verbal planning (Tower), no group differences between patients and control data were found (Table 3). However on most conditions of the Trail Making test (visual scanning, number sequencing, letter sequencing, number/letter switching) a significant difference in performance between the SCA-6 group and control data was demonstrated (effect sizes: $d = -0.68$, $-0.52$, $-0.45$ and $-0.56$ respectively). This cannot be explained by a difficulty with the motor aspect of the task as the motor speed subtest measure was comparable to that of the standardised normal data and only 2 patients

Table 2
Memory functioning of SCA6 group and comparison with control data

| Index scale | N | Mean (SD) | Statistical analysis (p-value) | Number impaired (%) |
|---|---|---|---|---|
| Auditory Immediate | 27 | 96 (17) | $t = -1.3$ (ns) | 7 (26%) |
| Visual Immediate | 27 | 99 (19) | $t = -0.31$ (ns) | 8 (30%) |
| Immediate Memory | 27 | 95 (20) | $t = -1.2$ (ns) | 8 (30%) |
| Auditory Delayed | 27 | 97 (16) | $t = -0.86$ (ns) | 5 (19%) |
| Visual Delayed | 27 | 99 (17) | $t = -0.16$ (ns) | 6 (22%) |
| Auditory Recognition Delayed | 27 | 101 (12) | $t = -0.24$ (ns) | 4 (15%) |
| General Memory | 27 | 99 (17) | $t = -0.30$ (ns) | 6 (22%) |
| Working Memory | 27 | Median = 104 | $W = 238$ (ns) | 1 (3%) |

Mean WMS-III Index Scores with one-tailed one-sample t-test (t) (parametrically distributed data) and one-tailed one sample Wilcoxon test (W) (non-parametrically distributed data) results to compare patient group performance with standardised control data with mean and median 100 and standard deviation 15 (ns denotes 'not significant'). Number and percentage of individually impaired patients also included (index score 15 points or more below the control mean).

Table 3
Executive functioning of SCA6 group and comparison with control data (D-KEFS)

| D-KEFS subtest | Subtest condition | N | Mean (SD) | Statistical analysis (p-value) | Number impaired (%) |
|---|---|---|---|---|---|
| Trail Making | Visual Scanning | 23 | 7.6 (4.1) | $t = -0.88$ ($< 0.01$)* | 9 (39%) |
| | Number Sequencing | 23 | 8.2 (4) | $t = -2.2$ ($< 0.05$)* | 9 (39%) |
| | Letter Sequencing | 23 | 8.4 (4) | $t = -1.9$ ($< 0.05$)* | 7 (30%) |
| | Number/Letter Switching | 23 | 8 (4) | $t = -2.4$ ($< 0.05$)* | 11 (48%) |
| | Motor Speed | 22 | Median = 11 | $W = 99$ (ns) | 2 (9%) |
| Verbal Fluency | Letter Fluency | 26 | 9.5 (4.1) | $t = -0.66$ (ns) | 8 (31%) |
| | Category Fluency | 26 | 9.5 (3.8) | $t = -0.62$ (ns) | 7 (27%) |
| | Category Switching Accuracy | 26 | 10 (3.1) | $t = 0.44$ (ns) | 5 (19%) |
| Design Fluency | Filled Dots | 22 | 11 (2.5) | $t = 1.5$ (ns) | 2 (9%) |
| | Empty Dots | 22 | Median = 9.50 | $W = 110$ (ns) | 4 (18.2%) |
| | Switching | 22 | 10 (3.3) | $t = 0.13$ (ns) | 5 (23%) |
| Colour/Word Interference | Colour Naming | 26 | 7.1 (4.3) | $t = -3.5$ ($< 0.01$)* | 13 (50%) |
| | Colour Word Reading | 26 | 7.6 (4.1) | $t = -2.9$ ($< 0.01$)* | 12 (46%) |
| | Inhibition | 25 | 7.2 (4) | $t = -3.4$ ($< 0.01$)* | 12 (48%) |
| | Inhibition/ Switching | 24 | Median = 9 | $W = 81$ (ns) | 8 (33%) |
| Card Sort | Free Sort | 25 | 9.8 (2.9) | $t = -0.20$ (ns) | 6 (24%) |
| | Free Sort Description | 25 | 9.2 (3.5) | $t = -1.2$ (ns) | 9 (36%) |
| | Sort Recognition | 25 | 7.8 (1.9) | $t = -5.6$ ($< 0.01$)* | 12 (48%) |
| | Initial Abstraction | 25 | Median = 9 | $W = 52$ ($< 0.01$)* | 10 (40%) |
| 20 Questions | | | | | |
| | Number of Questions Asked | 25 | 9.9 (3) | $t = -0.13$ (ns) | 5 (20%) |
| | Total Achievement | 25 | 9.9 (3.1) | $t = -0.19$ (ns) | 4 (16%) |
| Word in Context | Number Consecutively Correct | 26 | 9.5 (3.1) | $t = -0.88$ (ns) | 8 (31%) |
| Tower | Total Achievement | 25 | 9.6 (3) | $t = -0.66$ (ns) | 6 (24%) |

D-KEFS subtest mean scores with one-tailed one-sample t-test (t) (parametrically distributed data) and one-tailed one-sample Wilcoxon Test (W) (non-parametrically distributed data) results to compare patient performance with standardised control data with mean and median 10 and standard deviation 3 (ns denotes 'not significant', * denotes significance at the $p = 0.05$ level). Number and percentage of individually impaired patients also included (index score 3 points or more below the control mean).

Table 4
Non-verbal cognitive flexibility in SCA-6 group and comparison with control data Brixton test of Spatial Anticipation

| N | Mean (SD) | Statistical analysis (p-value) | Number impaired (%) |
|---|---|---|---|
| 27 | 91 (20) | $t = -2.5$ ($< 0.05$)* | 9 (33%) |

Error scores for SCA-6 group were converted to standard scores using age matched control data from Beilak et al. [3]. SCA-6 mean error standard score is presented with a one-tailed one-sample t-test result to compare patient performance with standardised control data (mean = 100, standard deviation = 15). (*denotes significance at the $p =$ 0.05 level). Number and percentage of individually impaired patients also included (index score 15 points or more below the control mean).

can be classified as having impaired performance. A significant deficit in performance was also noted in all but 1 (inhibition/switching) of the conditions of the Colour/Word Interference task (effect sizes: naming: $d = -0.8$; reading: $d = -0.66$ and inhibition: $d = -0.8$). In the recognition condition of the Card Sort task where the subject has to describe to the examiner how and why the cards have been sorted into the group presented, a significant performance deficit in the SCA-6 group was found (effect size: $d = -0.9$). In the 20 Questions task, the score of initial abstraction which is a measure of how well the subject uses their first question to eliminate the maximum possible number of objects was also found to be significantly lower than the standardised control data (effect size for nonparametric tests could not be calculated).

In a non-verbal task of spatial anticipation and cognitive flexibility, the Brixton test of Spatial Anticipation, a significant increase in the number of errors produced by the SCA-6 group as compared to healthy controls of the same age [3] was found (effect size: $d = -0.53$) (see Table 4).

### 3.2. Correlation analysis

Severity of motor impairment and duration of disease are both related to the degree of cerebellar damage. The relationships between these two indicators of degeneration and each cognitive test indices were investigated using Spearman's rank-order rho correlation coefficient (one-tailed).

A significant correlation between severity of motor impairment and all WAIS-III indices except Verbal Comprehension was demonstrated (see Table 5). Disease duration showed a significant negative correlation with Verbal Working Memory and Processing Speed indices. Disease duration yielded a significant negative relationship with all index scores of the WMS-III with the exception of the Auditory Recognition Delayed Index. No correlations between any of the WMS-III index scores and severity of motor impairment were found (see Table 6).

Table 5
Spearman's rho correlations (one-tailed) between WAIS-III index scores, severity of motor impairment and disease duration

| WAIS-III index scale | Severity of motor impairment | Disease duration |
|---|---|---|
| Verbal IQ | −0.48* | −0.22 |
| Performance IQ | −0.47* | −0.32 |
| Full Scale IQ | −0.49* | −0.34 |
| Verbal Comprehension | −0.21 | −0.65 |
| Perceptual Organisation | −0.46* | −0.25 |
| Verbal Working Memory | −0.52* | −0.37* |
| Processing Speed | −0.36* | −0.57* |

*denotes significance at the $p = 0.05$ level.

Table 6
Spearman's rho correlations (one-tailed) between WMS-III index scores, severity of motor impairment and disease duration

| WMS-III index scale | Severity of motor impairment | Disease duration |
|---|---|---|
| Auditory Immediate | −0.08 | −0.48* |
| Visual Immediate | −0.23 | −0.49* |
| Immediate Memory | −0.16 | −0.46* |
| Auditory Delayed | −0.07 | −0.37* |
| Visual Delayed | −0.15 | −0.40* |
| Auditory Recognition Delayed | −0.17 | −0.16 |
| General Memory | −0.15 | −0.41* |
| Working Memory | −0.31 | −0.46* |

*denotes significance at the $p = 0.05$ level.

Table 7 details the correlations between the executive function subtests (D-KEFS and Brixton Test of Spatial Anticipation) and the two indicators of cerebellar degeneration. Severity of motor impairment is negatively correlated with the switching and motor speed conditions of the Trail Making Test, the reading, inhibition and inhibition/switching conditions of the Colour/Word Interference test, the free sort and free sort description of the Card Sort test and finally with the Word in Context test. A significant negative relationship was demonstrated between disease duration and all conditions of the Trail Making test (with the exception of motor speed), two conditions of the Design Fluency test (empty dots and switching), all conditions of the Colour/Word Interference task and the Word in Context task.

## 4. Discussion

### 4.1. Current findings

This is the largest systematic assessment of cognitive function in a homogeneous group of patients with pure cerebellar atrophy. There was no evidence of a

Table 7
Spearman's rho correlations (one-tailed) between scores on tasks of executive function, disease duration and severity of motor impairment

| Subtest | Subtest condition | Severity of motor impairment | Disease duration |
|---|---|---|---|
| Trail Making | Visual Scanning | −0.31 | −0.56* |
| | Number Sequencing | −0.29 | −0.72* |
| | Letter Sequencing | −0.21 | −0.64* |
| | Number/Letter Switching | −0.61* | −0.50* |
| | Motor Speed | −0.37* | −0.34 |
| Verbal Fluency | Letter Fluency | −0.06 | −0.31 |
| | Category Fluency | −0.17 | −0.31 |
| | Category Switching Accuracy | −0.18 | −0.18 |
| Design Fluency | Filled Dots | −0.29 | −0.38 |
| | Empty Dots | −0.19 | −0.65* |
| | Switching | −0.27 | −0.62* |
| Colour/Word Interference | Colour Naming | −0.27 | −0.53* |
| | Colour Word Reading | −0.47* | −0.41* |
| | Inhibition | −0.54* | −0.52* |
| | Inhibition/ Switching | −0.54* | −0.51* |
| Card Sort | Free Sort | −0.46* | −0.30 |
| | Free Sort Description | −0.47* | −0.26 |
| | Sort Recognition | −0.15 | −0.23 |
| 20 Questions | Initial Abstraction | −0.32 | −0.20 |
| | Number of Questions Asked | 0.31 | 0.09 |
| | Total Achievement | 0.23 | 0.20 |
| Word in Context | Number Consecutively Correct | −0.39* | −0.43* |
| Tower | Total Achievement | −0.28 | −2.70 |
| Brixton Test of Spatial Anticipation | Number of Errors Made | −0.31 | −0.31 |

Results for both D-KEFS subtests and Brixton Test of Spatial Anticipation, *denotes significance at the $p = 0.05$ level.

generalised intellectual decline in the group as a whole. Hypothesised deficits in certain tests of executive functions and spatial cognition were confirmed. The SCA-6 subjects as a group were impaired specifically in tests of executive function that require verbal conceptual and abstract reasoning (card sort recognition and 20 Questions) and also in both verbal and non-verbal response inhibition and cognitive flexibility (Colour/Word Interference, Brixton test and Trail Making Task). Tests requiring problem solving, planning, verbal and spatial working memory and verbal and non-verbal fluency, as well as index scores related to all domains of memory performance were not significantly affected in this group. These findings are supported by a number of clinical studies of patients with cerebellar disorder where impaired performance on tests of set shifting, cognitive flexibility and response inhibition is consistently reported [22,23,39,42,60].

### 4.2. *Cognitive deficits are due to cerebellar deterioration rather than possession of the SCA-6 gene*

It is unlikely the mild cognitive deficits we have illustrated in this group are simply due to the presence of the abnormal gene defining the patient group. The cerebellum develops to a size comparable to healthy controls in SCA-6 [44]; in addition the significant inverse correlations for cognitive subtests requiring both a motor and non-motor response with severity of motor impairment and disease duration (as indicators of cerebellar degeneration) suggest the deficits we show here are due to the deterioration of the cerebellum in these patients. We have shown here that the correlations between motor deterioration and cognitive performance are dissociated from those between disease duration and cognitive performance (for example table 5 shows greater magnitude and significance of the correlation between motor deterioration and intelligence). We suggest that this reflects the fact that SCA-6 shows a homogeneous pattern of progression but heterogeneous rate of progression, thus making motor impairment a better index in the amount of cerebellar loss. Further studies are underway to collect data to allow a direct comparison between cerebellar grey matter volume changes and cognitive profile.

### 4.3. *Degeneration of the cerebellum in SCA-6 leads to mild executive dysfunction apparent in individual subjects*

In addition to assessing the effect of cerebellar disorder at the group level, the current study also assessed the prevalence of abnormal performance in individual patients. Cut-off scores of $\leqslant 85$ (WAIS-III, WMS-III and Brixton) and $\leqslant 7$ (D-KEFS) were used to define impairment in individuals. This represents a score $\geqslant -1$ standard deviation which is expected in 16% of the normal population [14,64,65]. A higher proportion of patients than would be expected in a normal population could be classed as impaired on most tasks of executive functions. More specifically a high proportion ($> 30\%$) of patients are impaired on tasks that require cognitive flexibility, inhibition of inappropriate response, both verbal and non-verbal concept abstraction and formation, and sequencing. Although no difference between control data and patient performance was demonstrated at the group level, Verbal Fluency is impaired in 19–31% of individual SCA-6 patients. The Verbal Fluency subtests did not display any relationship with the level of motor impairment suffered. However in a separate analysis using the dysarthria subscale of the motor impairment scale, a relationship between the extent of dysarthria and the performance of the letter and category fluency subtests was found, suggesting dysarthria may be involved in this impairment to some extent. Nevertheless the impairments at the individual level in this patient group reflect those at the group level, and support a role for the cerebellum in certain aspects of executive function.

There was no difference between the SCA-6 patients and the standardised normal data for the Tower test at the group level, contrary to findings of other studies [22,23,39,42]. However, 24% of individuals could be classed as impaired on this test. The Tower task is a good test of executive functioning, involving not only complex planning processes, but also inhibition of inappropriate response and maintenance of cognitive set. In an analysis of the number of rule violations in the Tower task, no difference again was found between the standardised normal data and the SCA-6 group data. Moreover only 7% of individuals were found to be impaired on this measure. It is possible that in the administration of the Tower test, the rules that the subject has to follow are re-enforced throughout the test: a written copy of the rules are presented to the subject with every new item throughout the test and when the subject first makes a mistake they are told that they have made an error. The absence of a group difference in this task, yet the presence of individual impairment, may underline the suggestion that the dysexecutive syndrome in SCA-6 is subtle and that difficulties with planning may occur as the disease progresses.

### 4.4. *Intelligence and memory deficits apparent in individual subjects*

A higher proportion of impaired patients than expected was found for Performance IQ and Full Scale IQ (a composite score that includes the Performance IQ score). There are two possible explanations for this. Firstly Performance IQ includes an assessment of novel problem solving tasks that are dependent on aspects of executive function such as non-verbal concept abstraction and formation (matrix reasoning) and sequencing (picture arrangement). As stated above, performance on tasks requiring these cognitive processes is impaired at both the group and individual level in the D-KEFS battery and Brixton Test of Spatial Anticipation; therefore it is possible that the higher proportion of individual impairment is a reflection of disruption of these cognitive processes rather than a general decline in intellectual ability. This is further supported by Verbal IQ being intact at both a group and individual level. Secondly some of the subtests that constitute Performance IQ are timed and require a motor response (e.g. block design and picture arrangement). The inverse correlations between Performance IQ and motor impairment suggest that the motor requirements of the test may contribute to the lower IQ measures in some individual patients.

The number of patients impaired on the index scales of Immediate Memory was also higher than expected. Immediate Memory deficits have been reported in earlier studies of cognition in SCA6 participants [18, 32,60], with preservation of Delayed Memory performance [18,20,32,60].

The data suggest that at the individual level, patients may exhibit executive functioning difficulties when tasks require cognitive switching, inhibition of inappropriate response, verbal and non-verbal concept formation and abstraction, sequencing and to some extent, verbal fluency. IQ will be intact with perhaps a slight deterioration of immediate memory processing as the disease progresses. These findings are supportive of previous studies of SCA-6 participants where deficits in set shifting [20,32,60], abstract reasoning [60] and spatial cognition [18,32,60] have been noted, along with deterioration of immediate memory processing [18,32, 60] and a reduction in verbal fluency [20,32,60].

### *4.5. Anatomical basis for the executive dysfunction*

Executive dysfunction is a classical manifestation of damage to the dorsolateral prefrontal cortex [46]. The contribution of the cerebellum to cognitive processes is hypothesised to be facilitated by cerebrocerebellar interconnections [15,43]. The terminations from the cortical projections are topographically organised in the cerebellum [33,51]: the cerebellar anterior lobe receives afferents from the motor and premotor cortices; while the posterior lobe, specifically crus I and II, receives afferents from the prefrontal cortex. This would suggest that in our group of patients where the degeneration progresses from anterior to posterior (see Fig. 1), executive functions modulated by the cerebellum via the frontocorticocerebellar connections, would be more vulnerable to cerebellar degeneration at the mid to later stages of the disease.

Without having functional imaging data for our patient group we cannot completely rule out abnormal activity (with intact structure) in other brain areas that could account for the cognitive impairments we have found in the current study. Kawai et al. [32] found evidence for reduced cerebral blood flow in prefrontal areas in a group of SCA-6 subjects. Moreover this hypoperfusion was correlated with impaired performance on tasks of visual memory, verbal fluency and rule shifting suggesting that cognitive impairment in SCA-6 may result from this prefrontal hypoperfusion. Crossed cerebrocerebellar diaschisis also cannot be ruled out as an underlying cause for the cognitive deficits in SCA-6 that we have shown in this study, as unilateral damage to the cerebellum has been shown to be accompanied by reduced blood flow in the contralateral basal ganglia and the frontoparietal cortex [6]. However, we suggest that the cerebellum is part of a network underlying executive function and damage to a single part of the network (such as exclusive Purkinje cell death in SCA-6) will disrupt the whole network leading to cognitive dysfunction. More research into brain activity in SCA-6 patients is warranted to discover how this network may be disrupted by damage to the cerebellum.

### *4.6. Comparisons with the Cerebellar Cognitive Affective Syndrome*

The CCAS predicts that patients with cerebellar disorder should develop a global cognitive disorder including linguistic deficits, visuospatial impairment, executive dysfunction and emotional and behavioural changes [52]. The present study does not support all of the aspects of the CCAS in a group of patients with a pure cerebellar degeneration. Difficulties in linguistic tasks such as naming and vocabulary, separate to the degree of dysarthria suffered by the patient are reported as an aspect of the CCAS. No difference from standardised norms was found in this study at either group or individual level for Verbal IQ and its comprising subtests, implying that non-motor language abilities are unaffected by the cerebellar degeneration in SCA-6.

Spatial cognition is reported to be significantly impaired in Schmahmann and Sherman's patients. With the exception of performance on the Brixton Test of Spatial Anticipation, we show no evidence that performance in other tasks involving a spatial component (i.e. visuospatial intelligence and memory tasks) was affected in this group of patients.

This study did not specifically test for personality and affect change; Schmahmann and Sherman report childlike and regressive behaviour in their patient group, which was not present in this study group. Patients' families were asked about changes in behaviour or mood during an interview before testing; neither patients nor their families reported any change in affect or personality and all behaved in an appropriate and cooperative manner throughout the assessment sessions.

Schmahmann and Sherman described problems with executive function, but to a more widespread extent than illustrated here. Our patients showed a mild dysexecutive syndrome in specific areas of executive function namely flexibility of thinking, inhibition, sequencing and in certain cases at the individual level, verbal fluency. These deficits occur to a large extent in the absence of intellectual decline in our patients, contrary to the general decrease in intellectual function predicted by the CCAS [52].

In the patient group described by Schmahmann and Sherman, those with anterior lesions exhibited fewer symptoms of the CCAS than those with more posterior lesions. As the degeneration in SCA-6 progresses in a stereotypical way from the superior-anterior to posterior-inferior cerebellum [8], it is possible that our patients show less cognitive disturbance than Schmahmann and Sherman's patients due to the more anterior nature of the degeneration in this disease. However, our cohort includes patients with advanced disease and severe global degeneration (see Fig. 1) assessed using measures that allow individual subject inference: these severely affected subjects show the same selective pattern of individual deficits as the group as a whole. One other factor to be taken into account in comparing the pattern of cognitive impairment shown here, with that described by the CCAS, is the plasticity of brain functions in this slowly progressive disorder, as opposed to the immediacy of deficits following acute cerebellar insult in a number of previous studies e.g. [17,23,47, 49,56].

### 4.7. *The generalised integrative role of the cerebellum*

In motor processing, the cerebellum is thought to play an important role in the integration of external, proprioceptive, sensory input and internal, motor planning information from the cortex, resulting in continuous, smooth movement [2,24]. Parsons and colleagues [45] suggest this integrative role is the primary function of the cerebellum rather than one of motor control *per se*. This suggestion was based on findings of dentate nucleus activation during sensory stimulation of the fingers, but not during finger movement. This apparent integrative role of the cerebellum may be as crucial to cognitive processes as it is for normal motor function [4]: Ito [27] describes the role of the cerebellum as an encoder of internal models for the smooth control of both motor and higher cognitive processes. This theory predicts that in the same way that damage to the cerebellum disrupts, rather than destroys, voluntary movement [25,26], cognitive processes are disrupted, becoming inflexible and uncoordinated; producing problems in tasks requiring flexibility of thought and organisation of response.

This parallel disruption of cognitive and motor processes is the basis for the 'dysmetria of thought' hypothesis put forward by Schmahamann [50]. This hypothesis states that the cerebellum regulates the speed, accuracy, appropriateness and consistency of thought. Damage to the cerebellum leads to disruption of these processes, causing erratic attempts to correct behaviour and thought. The disruption in cognitive flexibility, inhibition and integration of information found in our patients may reflect this disturbance in the underlying regulatory and correctional function of the cerebellum for both motor and cognitive control.

## 5. Conclusions

We have demonstrated that the cognitive profile of SCA-6 at the group level is characterised by specific dysfunction in processes requiring cognitive flexibility, response inhibition, sequencing and the integration of spatial information, in the context of intact intellectual and memory functions. A similar profile of significant deficits can be seen in individual patients. These data provide support for some aspects of the CCAS [52] and are consistent with a model of cerebellar disorder, which highlights the role of the cerebellum in the correction and coordination of cognitive processes that parallels this structure's role in motor control.

## Acknowledgements

The authors would like to thank the participants of this study, without whose continuing support it would not be possible to perform such research. This project was funded by Ataxia UK. TDG and MG are funded by the Wellcome Trust (UK).
